# The Role of Toll-Like Receptor Signaling in the Progression of Heart Failure

**DOI:** 10.1155/2018/9874109

**Published:** 2018-02-08

**Authors:** Lili Yu, Zhiwei Feng

**Affiliations:** ^1^School of Basic Medical Sciences, Xinxiang Medical University, Xinxiang, Henan 453003, China; ^2^Pennington Biomedical Research Center, Louisiana State University, Baton Rouge, LA 70808, USA; ^3^Henan Key Laboratory of immunology and Targeted Drugs, Xinxiang, Henan 453003, China; ^4^Henan Collaborative Innovation Center of Molecular Diagnosis and Laboratory Medicine, Xinxiang, Henan 453003, China

## Abstract

Medical systems worldwide are being faced with a growing need to understand mechanisms behind the pathogenesis of heart failure (HF) that is considered as a leading cause of morbidity and mortality around the world. Elevated levels of inflammatory mediators have been identified in patients with HF, which are primarily manifestations of innate immune responses mediated by pattern recognition receptors (PRRs). Toll-like receptors (TLRs), which belong to PRRs, are subjected to the release of pathogen-associated molecular patterns (PAMPs) and damage-associated molecular patterns (DAMPs) to generate innate immune responses. More and more emerging data indicate that TLR signaling pathway molecules are involved in the progression of HF. Herein, we present new data with regard to the activation of TLRs in the failing heart, focusing on TLR2, TLR3, TLR4, and TLR9, and suggest the potential use of TLRs in target therapy.

## 1. Introduction

Heart failure (HF) is a complex clinical syndrome and occurs during structural or functional impairment of ventricular filling or ejection of blood in the heart that fails to pump sufficiently to maintain blood flow that meets the body's needs. HF is a leading cause of morbidity and mortality worldwide, and it increasingly affects millions of people [1, 2]. There are three types of HF, the left-sided HF, right-sided HF, and congestive HF, according to the classification of the American Heart Association. There are two kinds of left-sided HF: one is systolic HF, which means that the left ventricle loses its ability to contract normally causing the inability of the heart to pump with enough force to push enough blood into circulation; the other is diastolic HF, which means that the left ventricle loses its ability to relax normally causing the inability of the heart to be properly filled with blood during the resting period between each beat. Right-sided HF usually occurs when the right ventricle loses its ability to be filled with or to inject blood properly. Congestive HF describes the condition that when blood flowing out of the heart is slower than normal, blood returning to the heart through the veins backs up, causing congestion in the body's tissues, including the arms, legs, ankles, feet, and lungs.

HF is caused by many conditions that damage the heart muscle. Ischemic heart disease (IHD) is the number one leading cause of HF according to epidemiological studies. In clinical trials, HF has been ascribed to IHD in about 70% of patients [3]. Other common causes are involved in the progression of HF, including dilated cardiomyopathy (DCM), cardiomyopathy of an unknown cause, hypertension, atrial fibrillation, infection, excess alcohol use, metabolic syndrome, atherosclerotic disease, myocarditis, and cardiomyopathy due to inflammation [1, 4].

Growing evidence supports that inflammation has been implicated in the pathogenesis of HF [4, 5]. Inflammation of the heart may cause HF in about 10% of cases of initially unexplained cardiomyopathy [6, 7]. A variety of infectious organisms, as well as toxins and medications, most often postviral in origin, may cause myocarditis. The link between HF and inflammation was first recognized in 1990 by Levine et al., who reported elevated levels of tumor necrosis factor *α* (TNF*α*) in patients with HF with reduced ejection fraction [8]. Numerous studies have demonstrated that patients with HF exhibited raised circulating levels of other inflammatory cytokines, such as interleukin- (IL-) 1*β* and IL-6, and several chemokines, including monocyte chemoattractant peptide- (MCP-) 1, IL-8, macrophage inflammatory protein- (MIP-) 1*α*, and galectin-3 [9–14]. These data suggest that increased systemic levels of inflammatory cytokines in patients with HF may reflect important pathogenic mechanisms. The systemic metabolic disorders induce subcellular component abnormalities, such as oxidative stress, mitochondrial dysfunction, endoplasmic reticulum (ER) stress, and impaired calcium handling, leading to impaired myocardial relaxation [15]. In addition to myocardium itself, several tissues and cells, including leukocytes, platelets, tissue macrophages, and endothelial cells, can contribute to this inflammation. In the advanced stage, increased subcellular component abnormalities, inflammatory cell infiltration, neurohumoral activation, and their vicious cycle induce cardiomyocyte injury and death and cardiac fibrosis, resulting in impairment of both diastolic and systolic functions, leading to HF [15, 16]. In addition, these inflammatory mediators may serve as relevant markers of disease severity and HF prognosis [17–21]. Importantly, the association between HF and markers of inflammation was observed for both reduced and preserved ejection fraction cohorts [22].

In short, inflammation plays a critical role in myocardial ischemia and the development of HF. More and more evidence is emerging to explore the function of Toll-like receptors (TLRs) in inflammation-caused HF. In this review, we will focus on the roles of TLRs in the progression of HF, updating recent findings.

## 2. Pattern Recognition Receptors (PRRs)

Inflammatory signaling in cardiomyocytes usually occurs as an early response to myocardial injury. Innate immune activation is a key pathogenic mechanism in HF. Mann's study showed the close correlation between innate immunity and HF. Gene arrays from explanted hearts from patients with ischemic cardiomyopathy (ICM), idiopathic DCM, viral cardiomyopathy, and nonfailing hearts show the different expressions of innate immune genes in the failing heart compared to nonfailing hearts, and elevated levels of inflammatory mediators have been identified in patients with a failing heart; this observation indicates the possibility of activation of the innate immune system in failing hearts [11, 23].

Cardiac innate immune responses, which are essential for homeostatic responses and tissue repair, are initiated by germline-encoded PRRs, which include TLRs, retinoic acid-inducible gene-I-like receptors, nucleotide-oligomerization domain-like receptors (NLRs), C-type lectin receptors (CLRs), and absent-in-melanoma 2 receptors [24, 25]. The strategy of PRR recognition is based on the detection of constitutive and conserved pathogen-associated molecular patterns (PAMPs), which include bacterial carbohydrates, nucleic acids, bacterial peptides, peptidoglycans, lipoteichoic acids, *N*-formylmethionine, lipoproteins, fungal glucans, and chitin. PRRs can also recognize endogenous stress signals called damage-associated molecular patterns (DAMPs), including uric acids, extracellular ATP and other compounds [26]. Recently, it has become clear that cardiac PRRs also recognize the molecular patterns of endogenous host material released by dying or injured myocardial cells [11]. Cells that die by accidental necrosis, necroptosis, or secondary apoptosis release their cytosolic contents into the extracellular space, thereby initiating inflammatory responses through engagement of an ensemble of extracellular or intracellular PRRs.

## 3. The TLR Signaling Pathway

TLRs belong to PRRs, which are involved in the inflammatory responses during HF [27]. To date, 13 TLRs have been identified in mammals, with 10 in humans and 12 found in mice; TLRs are type I transmembrane glycoproteins comprising extracellular, transmembrane, and intracellular domains [28, 29]. TLRs are classified into two main groups according to their subcellular localization; TLR1, TLR2, TLR4, TLR5, TLR6, and TLR11 are expressed on the plasma membrane, whereas TLR3, TLR7, TLR8, and TLR9 are found in endosomes [30, 31].

TLRs usually function as dimers for PAMP detection. Individual TLRs differentially recruit members of a set of Toll/IL-1 receptor (TIR) domain-containing adaptors [32]. Five TIR domain-containing adaptors have been identified, namely, myeloid differentiation factor 88 (Myd88), Myd88 adaptor-like protein, TIR domain-containing adaptor protein inducing interferon (IFN)-*β*-mediated transcription factor (TRIF), TRIF-related adaptor molecule (TRAM), and a sterile *α*- and armadillo motif-containing protein [33, 34]. Based on specific adaptors recruited to TLRs, TLR signaling can be divided into two general pathways, namely, the Myd88-dependent and Myd88-independent pathways. Except for TLR3, all TLRs interact with the adaptor protein Myd88. TLR3 uses TRIF as the adaptor protein belonging to Myd88-independent pathways, whereas TLR4 triggers both the Myd88-dependent and Myd88-independent pathways [35]. These signaling pathways activate numerous transcription factors, such as nuclear factor-*κ*B (NF-*κ*B) and interferon (IFN) regulatory factors (IRFs), and subsequently induce the production of proinflammatory cytokines and IFNs, respectively [36].

**Figure 1:**
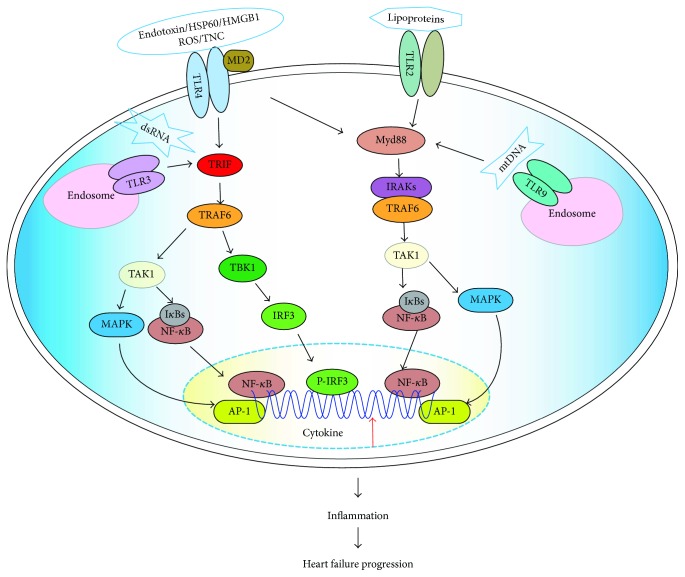
Activation of TLRs in heart cells by PAMPs and DAMPs during heart failure. Heart cells express a variety of TLRs, mainly, TLR2, TLR3, TLR4, and TLR9. DAMP and PAMP molecules, which include endotoxin, HSP60, HMGB1, ROS, TNC, lipoproteins, virus RNA, and mtDNA, are involved in HF. Lipoproteins have been reported to activate TLR2. Endotoxin, HSP60, HMGB1, ROS, and TNC have been demonstrated to activate TLR4. dsRNA can be recognized by TLR3. TLR9 can recognize mtDNA to induce immune responses. There are two pathways for TLR signaling, including the Myd88-dependent and Myd88-independent signaling pathways. TLR2 and TLR9 utilize the Myd88-dependent pathway. TLR3 uses the Myd88-independent pathway. TLR4 employs both Myd88 and TRIF as adaptor proteins. Note that TLR3 and TLR9 are predominately located within endosomes. In the Myd88-dependent signaling pathway, stimulation of TLR triggers association of My88, which in turn recruits the IRAK family, and subsequently, TRAF6 is also recruited to the receptor complex by associating with phosphorylated IRAKs. Ubiquitylation of TRAF6 induces the activation of TAK1, which phosphorylates both MAPK kinases and the IKK complex consisting of IKK-*α*, IKK-*β*, and IKK-*γ*. The IKK complex then phosphorylates I*κ*B, which is then ubiquitylated and subsequently degraded. This result allows NF-*κ*B to translocate to the nucleus and induce the expression of its target genes. TRIF play an essential role in the Myd88-independent pathway through TLR3 and TLR4. TRIF interacts with TRAF6, which activates TBK1 and IKK-*ε* for phosphorylation of the transcription factor IRFs. TRIF can also promote NF-*κ*B activation. TRIF recruits TRAF6 and activates TAK1, which in turn activates the NF-*κ*B and MAPK pathways. These signaling pathways result in the expression of cytokines. Inflammation induces cell injury and death, resulting in cardiac dysfunction and HF progression.

The Myd88-dependent pathway is initiated via Myd88 after TLR activation [37]. Afterwards, the death domain of Myd88 recruits IL-1 receptor-associated kinase 4 (IRAK4) and activates one of other IRAK family members, that is, IRAK1 or IRAK2. Then, IRAKs dissociate from the My88-IRAK complex and activate TNF receptor-associated factor 6 (TRAF6), which interacts with transforming growth factor-*β*-activated kinase 1 (TAK1), TAK1-binding protein 1 (TAB1), and TAB2 [33]. TAK1 then activates the complex of inhibitory *κ*B (I*κ*B) kinase *α* (IKK*α*)/IKK*β*/IKK*γ* and induces I*κ*B phosphorylation. Phosphorylated I*κ*B dissociates from the complex and activates the transcription factor NF-*κ*B. The activated NF-*κ*B translocates into the nucleus and induces the expression of various proinflammatory cytokines. In addition to the activation of the IKK complex, TAK1 can activate the mitogen-activated protein kinase (MAPK) signaling pathway, including the extracellular signal-regulated kinase pathway, c-Jun N-terminal kinase pathway, and p38 pathway. The MAPK signaling pathway can activate the transcription factor activator protein-1 (AP-1). Activation of NF-*κ*B and AP-1 contributes to the expression of proinflammatory cytokines, such as IL-6, IL-1, and TNF*α* (Figure 1).

Also known as the TRIF-dependent pathway, the Myd88-independent pathway can lead to activation of IRFs and NF-*κ*B. This pathway is initiated by TRIF and TRAM. TRAM is a particular adaptor connecting TLR and TRIF. After recruitment, TRIF interacts with TRAF6, which activates TRAF family member-associated NF-*κ*B activator-binding kinase 1 (TBK1) and IKK-*ε* for phosphorylation of IRFs. Activated IRFs translocate into the nucleus to induce the production of IFNs. In another type of signaling, TRIF can promote NF-*κ*B activation. TRIF recruits TRAF6 and activates TAK1, which in turn activates NF-*κ*B and MAPK pathways. Activation of NF-*κ*B and AP-1 contributes to the expression of proinflammatory cytokines, whereas the activation of IRF3 contributes to the expression of interferon (Figure 1).

## 4. TLR Signaling and HF

Recently, increasing evidence indicated involvement of innate immune activation mediated by myocardial TLRs in HF [12]. TLRs are expressed in various types of heart cells, including endothelial cells, smooth muscle cells, and cardiomyocytes [38]. Relative expression levels for TLR mRNAs in the human heart follow the order TLR4 > TLR2 > TLR3 > TLR5 > TLR1 > TLR6 > TLR7 > TLR8 > TLR9 > TLR10 [11, 39]. These TLRs not only present different expressions but also perform different functions in the development of HF. Next, we will discuss several important TLRs, including TLR2, TLR3, TLR4, and TLR9, which are associated with HF (Figure 1, Table 1).

### 4.1. TLR2

TLR2 is located on the cell surface, and along with TLR1 or TLR6, it recognizes a wide variety of PAMPs, including lipoproteins, peptidoglycans, lipoteichoic acids, zymosan, mannan, and mucin [37]. A clinical study shows that there is a possible immunological role for lipoproteins in chronic HF [40].

TLR2 plays a central role in the pathogenesis of diverse heart disorders and is upregulated in doxorubicin-reduced DCM and HF [41]. An early report showed that TLR2 was expressed in cardiomyocytes, was a participant in responses of these cells to oxidative stress, and was a major contributor to the pathogenesis of cardiac dysfunction [42]. Vascular endothelial cells also express high levels of TLR2 on stimulation of inflammatory cytokines, suggesting that TLR2 can also contribute to endothelial cell-related inflammation [43]. In the therapeutic study, Ma et al. demonstrated that blockage of TLR2 reduced mortality and attenuated doxorubicin-induced cardiac dysfunction and inhibition of TLR2 showed a potential role for the treatment of DCM [41].

In a mouse model, TLR2 was involved in cardiac remodeling after myocardial infarction (MI), and preservation of cardiac function, increased survival rate, and attenuation of myocardial fibrosis after MI in *TLR2 KO* mice were observed [44]. Adverse ventricular remodeling following cardiac injury is a key determinant of HF. Other TLR2 studies were accompanied with TLR4. Activation of TLR2 and TLR4 worsened ischemic injury to the heart and brain of animal models of MI and stroke [45]. A study pointed out that TLR2 and TLR4 influence autonomic regulation of heart rate, and mice lacking TLR2 or TLR4 exhibited reduced basal heart rate [46].

Moreover, increased expression and signaling by TLR2 have been found to contribute to the activation of innate immunity in injured myocardium, indicating that TLR2 can promote myocardial inflammation in HF [44]; however, another study indicated that TLR2 expressions in patients with chronic HF are similar compared with that in the control group [47]. In conclusion, evidence is shown that inhibition of TLR2 reduces the progression of HF (Table 1).

In terms of inhibiting TLR2 signaling, antibodies are designed. T2.5 is one of the anti-TLR2 antibodies with therapeutic potential. Moreover, T2.5 was found to prevent angiotensin II-induced cardiac fibrosis through suppressing macrophage recruitment and inflammation in the heart [48].

### 4.2. TLR3

TLR3 is localized in endosomes and recognizes viral double-stranded RNA (dsRNA), small interfering RNA, and self-RNA that is derived from damaged cells [49].

Few studies have discussed virus-caused inflammation related to TLR3 in HF. Enterovirus-induced myocardial injury can lead to severe HF. TLR3 plays an important role in the initiation of innate antiviral responses. Hardarson et al. [50] reported that *TLR3 KO* mice were more susceptible for encephalomyocarditis virus infection and featured a higher viral load in the heart and that TLR3 was involved in mediating protection against virus-induced myocardial injury.

Another research by Wang et al. reported that *TLR3*-deficient neonatal hearts exhibited impaired cardiac functions and larger infarct size after MI compared to control, which indicated that TLR3 is also related with HF [51]. Similarly, Chen et al. reported that TLR3 signaling was involved in MI and extracellular RNA released during myocardial ischemia-reperfusion (I/R) injury, which may contribute to myocardial inflammation [52].

TLR3 primarily protects the heart against viral infection; however, TLR3 also mediates inflammatory effects that may exacerbate heart damage (Table 1).

### 4.3. TLR4

All known human TLRs have been detected in the heart and most importantly, TLR4, whose levels are the highest compared with other TLRs in the heart [23]. TLR4 plays a critical role in myocardial inflammation, including myocarditis, MI, myocardial I/R injury, HF, aortic valve diseases, atherosclerosis, and hypertension [27, 53].

TLR4 is located at the plasma membrane, where it responds to its ligands and triggers a series of inflammatory signaling pathways [54]. TLR4 is activated by lipopolysaccharide (LPS), with the cofactors, such as cluster of differentiation 14, myeloid differentiation factor 2 (MD2), and lipopolysaccharide- (LPS-) binding protein [55, 56]. Increased levels of bacterial LPS have been demonstrated in HF [57]. Endotoxin is an LPS constituent of the outer membrane of most Gram-negative bacteria. The endotoxin can bind to TLR4/MD2 complexes, which cause subsequent inflammation, and has been implicated in the development and progression of atherosclerosis and subsequent coronary artery disease and HF [58]. Another report also showed that the cardiac function in *TLR4*-deficient mice was not affected following septic shock or myocardial ischemia [59].

TLR4 can also recognize exogenous ligands, such as the fusion proteins from respiratory syncytial virus and glycerophosphatidylinositol anchors from parasites [31]. In a mouse model, Riad et al. [60] reported that coxsackievirus infection with *TRIF*-deficient mice can lead to the induction of severe HF and 100% mortality, displaying TLR4-dependent suppression of antiviral cytokine IFN-*β*. By contrast, coxsackievirus infection increased the cardiac levels of IL-1*β* and IL-18 in WT mice but not in *TLR4*-deficient ones, and TLR4 deletion may protect these animals from HF [61]. From a perspective, it is likely that the role of TLR4 is indistinct with regard to protecting the heart against viruses.

Some endogenous ligands, such as heat shock protein (HSP), high-mobility group box 1 (HMGB1), reactive oxygen species (ROS), and extracellular matrix components, can be recognized by TLR4 [31, 62]. Some of these ligands are associated with HF. HSP60 is doubly expressed in end-stage HF and presents abnormal trafficking to the cell surface, which may be an early trigger for myocyte loss and the progression of HF [63]. HMGB1 has been established as an important mediator of myocardial inflammation and is associated with the progression of HF. The study of Volz et al. [64] showed that HMGB1 plasma concentration was elevated in HF and correlated with disease severity in patients with HF. ROS can modify membrane components and can cause the release of factors that interact with and activate TLR4 to induce cardiomyocyte apoptosis and HF [10, 65]. Tenascins represent a family of four multimeric extracellular matrix glycoproteins [66]. Serum level of tenascin C (TNC) correlates with the severity of HF [67]. Maqbool et al. [68] reported that TNC can stimulate TLR4 to upregulate the expression of IL-6, contributing to the worsening and progression of HF.

Doxorubicin-induced systemic inflammation is driven by upregulation of TLR4 and endotoxin leakage [69]. TLR4 is upregulated in doxorubicin-induced DCM and HF like TLR2. But unlike the role of TLR2, Ma et al. showed that TLR4 played a distinct function in the progression of doxorubicin-induced DCM and blockage of TLR4-exacerbated cardiac dysfunction and fibrosis by amplifying inflammation [41].

Liu et al. [70] pointed that the expression, ligand-binding capacity, and proinflammatory function of TLR4 were upregulated in the cardiomyocytes isolated from the long-term MI, promoting inflammation and exacerbating HF. TLR4 not only is expressed in cardiomyocytes but is also a major feature of activated monocytes and substantially increases in response to DAMPs. Similar to TLR2, TLR4 was expressed at high levels in vascular endothelial cells; this finding suggests contribution of endothelial cell-related inflammation [43]. Peripheral monocytosis may affect the development of HF after acute MI (AMI). Activated TLR4 in monocytes plays an important role in the synthesis of proinflammatory cytokines. Activation of TLR4 through Cardiomyocytic inflammatory reaction was associated with HF after AMI [26].

TLR4 also has a proinflammatory role in murine myocardial I/R injury. In one study, *TLR4*-deficient mice sustained smaller infarctions and exhibited less inflammation after myocardial I/R injury [71]. Another study showed that inhibition of TLR4 in an in situ murine model significantly reduced I/R injury and markers of inflammatory response [72].

Studies have shown that TLR4 expression increases in the hearts of patients with advanced HF [73, 74]. Other studies indicated that unstimulated monocyte TLR4 expression was significantly higher in patients with chronic HF compared to controls and upregulation of monocyte TLR4 may contribute to pathophysiology of chronic HF [47]. TLR4 is associated with deleterious inflammatory effects that exacerbate heart damage, and inhibition of TLR4 reduces the progression of HF (Table 1).

Pharmacological blocking of TLR4 by different molecules is influenced. Statins are among the early-developed drugs with newly discovered inhibitory activity on TLR4 signaling. Among the statin family, fluvastatin, simvastatin, and atorvastatin, all have shown potent inhibitory activity on TLR4 and subsequent inflammatory pathways to reduce inflammation in vascular systems [48]. Another molecule eritoran, the antagonist of TLR4, is very helpful. Inhibition of TLR4 with eritoran can attenuate myocardial ischemia-reperfusion injury [75] and the development of cardiac hypertrophy [76]. There is a new finding of radioprotective 105 kDa protein RP105, which is a regulator of TLR4 and critical therapeutic target, which can protect against I/R injury via suppressing TLR4 signaling pathways in a rat model [77, 78]. Ghrelin is another candidate for suppression of TLR4 signaling and has protective effects against inflammation in a mouse model of myocardial I/R injury via the TLR4 pathway [79].

### 4.4. TLR9

TLR9 was first identified as a TLR-recognizing cytosine-phosphate-guanine (CpG) which repeats within microbial DNA and is expressed in the myocardium [80, 81]. TLR9 mainly signals through the Myd88-dependent pathway and stimulates NF-*κ*B and downstream signaling. Emerging evidence has shown the involvement of TLR9 in HF.

Stimulation with bacterial DNA or CpG-rich DNA can induce myocardial inflammation and reduce cardiomyocyte contractility through TLR9 [82]. Another research on a mouse model with polymicrobial sepsis indicated that *TLR9 KO* mice showed significant reduction in cardiac inflammation and sustained heart function, indicating that TLR9 promotes cardiac inflammation and HF during polymicrobial sepsis [83].

Mitochondrial DNA (mtDNA) is similar to bacterial DNA and may contain high contents of CpG that activates TLR9 [84, 85]. Recent data has demonstrated that mtDNA was a DAMP that activated TLR9 [86–88]. Extracellular mtDNA activates NF-*κ*B via TLR9 and can induce cell death of cardiomyocytes [89]. Pathophysiological significance of TLR9 in HF has been studied. Oka et al. [86] studied that the mtDNA that escaped from autophagy cell autonomously leads to TLR9-mediated inflammatory responses in cardiomyocytes and can induce myocarditis and DCM. DNase II is an acid DNase found in lysosomes and plays an important role in preventing pressure overload-induced HF. Inhibition or ablation of TLR9 attenuated the development of cardiomyopathy in DNase II-deficient mice. *TLR9 KO* mice showed improved pressure overload-induced cardiac dysfunction and inflammation [86]. On the contrary, Velten et al. [90] indicated that pretreatment with synthetic TLR9 ligand 1668-thioate attenuated cardiac hypertrophy following pressure overload and delayed the cardiac function loss, which resulted in a prolonged preservation of left ventricular function. A similar study showed another synthetic agonist of TLR9 that activated the phosphoinositide 3-kinase/protein kinase B signaling pathway and attenuated pathological cardiac hypertrophy and HF [91]. All these data have shown that altered TLR9 signaling influences the progression of HF although the results reflect some differences in experimental models.

Other studies showed that TLR9 plays an important role in diastolic HF. The sacro/endoplasmic reticulum Ca^2+^ ATPase (SERCA) is the nodal protein that governs active diastolic function [92]. Cardiomyocyte-specific deletion of SERCA2a leads to diastolic HF [93]. Dhondup et al. [94] reported that in a mouse model with diastolic HF caused by cardiomyocyte-specific deletion of SERCA2a, sustained activation of TLR9 caused cardiac and systemic inflammation and deterioration of SERCA2a depletion-mediated diastolic HF. In another diastolic HF mouse model induced by cardiomyocyte-specific deletion of SERCA2a, TLR9 depletion in those models reduced the survival rate compared with that of the *SERCA2a* KO control mice; this finding indicates the salutary role of TLR9 in some subsets of HF [95]. These studies suggest a link between systemic TLR9 activation and diastolic HF.

In a clinical study, Ye et al. [96] discovered increased plasma-derived exosomes in patients with chronic HF compared with healthy controls and demonstrated that plasma-derived exosomes carry mtDNA, which can trigger an inflammatory response via the TLR9-mediated NF-*κ*B pathway. Another study showed elevated plasma levels of mtDNA from patients with HF, and this condition was associated with increased mortality [97]. Interestingly, TLR9 is only expressed to a small amount in the human heart compared with other TLRs [11, 39] but does seem to play an important role in HF. This is a little contradiction. Actually, mitochondria are recognized as a key player in cardiomyocyte cell death after myocardial infarction and cardiomyopathies. TLR9 is very important for the recognition of mtDNA in mitochondria, and that may be the key point. These findings indicate that the TLR9 signaling pathway is involved in inflammatory responses and the pathogenesis of HF (Table 1).

### 4.5. The Downstream Molecular Pathway of TLR Signaling

TLR signaling pathway downstream molecules are involved in the HF progression. Myd88 is central to the signaling of most of the TLRs and receptors of the IL-1 family. One study pointed out that Myd88 deletion can protect mice from the progression of acute myocarditis to end-stage HF [98]. Other two studies showed that Myd88-mediated inflammatory signaling leads to CaMKII oxidation, cardiac hypertrophy, and death after MI and blockade of Myd88 with ST2825 or IMG2005 prevents left ventricular dilation and hypertrophy after acute MI [99, 100]. Myd88 could recruit the IRAK family member IRAK4, and deletion of IRAK4 has favorable effects on survival and left ventricular remodeling after MI [101]. Negative regulation of inflammatory signaling involves activation of distinct pathways in various cell types involved in cardiac repair. IRAK-M exerts its anti-inflammatory actions by inhibiting TLR/IRAK-1-dependent signaling in macrophages. Genetic IRAK-M loss was associated with accentuated inflammation and increased dilative remodeling following infarction [102, 103]. The IKK and its downstream target, NF-*κ*B, are regulators of inflammation and are activated in cardiac disorders [104, 105]. Maier et al.'s study showed that cardiomyocyte-specific IKK/NF-*κ*B activation induced reversible inflammatory cardiomyopathy and HF [106]. Frantz et al.'s study pointed out that deletion of the NF-*κ*B subunit p50 in mouse improved early survival and reduces left ventricular dilatation after MI; these findings indicate that NF-*κ*B may therefore be an attractive target for HF treatment [107]. The chemokine MCP-1, a downstream molecule of TLR signaling, has been considered as one of the biomarkers of HF [108].

## 5. Other PRRs and HF

NLRs act as cytosolic sensors to intracellular PAMPs and DAMPs. The human NLR family includes 22 members, most of which share a conserved tripartite structure consisting of an N-terminal caspase recruitment domain (CARD) or pyrin domain, a central nucleotide-binding domain with NTPase activity, and a C-terminal leucine-rich repeat domain that mediates ligand sensing [109]. NLRP3 belongs to the NLR family, together with apoptosis-associated speck-like protein containing a CARD protein, and forms the NLRP3 inflammasome. This inflammasome represents a complex of intracellular interaction proteins that trigger maturation of proinflammatory cytokines IL-1*β* and IL-18 by caspase-1 to initiate the inflammatory responses [110, 111]. The NLRP3 inflammasome signaling effector, caspase-1, is upregulated in murine and human failing hearts [112]. *NLRP3 KO* in cardiac-specific calcineurin transgenic mice resulted in DCM, reduced proinflammatory cytokine maturation and cardiac inflammation, and improved systolic performance [110]. TLR signaling is important for inflammasome priming, and without priming, NLRP3 activation may be insufficient for inducing cardiac dysfunction [113, 114]. NLRP3 inhibition has been shown to be protective for cardiac function after ischemic injury (AMI) and nonischemic injury (doxorubicin treatment) in mice [115]. Downstream proinflammatory cytokine IL-18 is being considered as a therapeutic target in acute MI and HF [116]. The Canakinumab Anti-inflammatory Thrombosis Outcomes Study (CANTOS) trial, using a blocker of IL-1*β*, has shown good results for the anti-inflammatory therapies in AMI and HF [117]. All these data show that NLRP3 inflammasome signals play an important role in modulating inflammation that affects HF progression. Another NLR protein, NLR family CARD domain-containing protein 4 (NLRC4), has been observed in heart diseases, and this study showed that NLRC4 inflammasome was hyperactivated by mitochondrial DNA in cardiomyocytes in a type 2 diabetes mouse model after MI [118].

CLRs are calcium-dependent carbohydrate-binding receptors that contain one or more C-type lectin-like domains. CLRs form a large family that recognizes a diverse array of structurally unrelated molecules. CLRs and CLR-related signaling molecules are constitutively expressed in human and murine hearts [119]. Expressions of CLRs and CLR-related signaling molecules in a healthy heart support the possible expression of CLRs in cardiomyocytes; however, additional work is needed to fully define the functions of these proteins.

## 6. Conclusion and Prospective

Inflammation has been widely accepted to play an important role in the physiological and pathological mechanisms of cardiac function and dysfunction. Inflammation is required for host defense against damage and tissue repair. However, excessive chronic myocardial inflammation is reported to induce severe damage to the myocardium and cause HF. The role of the innate immune system in the pathogenesis of heart diseases has been an area of particular focus; targeting innate immune molecules in experimental models can variously attenuate disease progression and injury and promote healing [12].

In this review, we described some recent advances in our understanding of the role of TLR receptors in HF. The provided data linking TLR signaling to HF is still being accumulated at the time of writing. TLR signaling pathway modulates much broader regulation of inflammatory mediators and acts as an important upstream mechanism for activating inflammatory signaling. Hence, target of TLR singnaling molecular in HF may offer a reliable therapeutic approach. Accordingly, various therapeutic agents for inhibiting TLR signaling have been developed to control excessive inflammation [48].

However, few issues remain unanswered. For instance, (1) the mechanism of TLR activation remains unelucidated. More ligands must still be identified in the failing heart; (2) regulation of TLRs in the failing heart also requires further research. Better understanding of these questions will potentially generate a novel therapy for preventing or slowing of the development and progression of HF. Moreover, targeting specific TLR pathways may supply smart strategies for patients with HF. However, at present, our knowledge of the role of TLR signaling is still too insufficient to support the evaluation of this therapy in clinical trials. Although in some models, the role of TLRs is to protect the heart, the expression of TLRs within the heart is often associated with inflammation that leads to increased cell apoptosis, cell necrosis, and tissue damage. On the other side, whether blocking these TLR receptors with antibodies or small molecule inhibitors could prevent the development of a heart failure phenotype needs further identification in animal models.

## Figures and Tables

**Table 1:** 

TLR	Expression	Related pathogenesis of HF	Therapy direction
TLR2	Second highest	Doxorubicin-induced DCM; MI; contribute to myocardial inflammation; similar expression in patients with chronic HF	Inhibition of TLR2 is beneficial for the progression of HF; blocking molecular T2.5 antibody

TLR3	Third highest	Protected virus-induced myocardial injury; MI; contribute to myocardial inflammation	Unclear

TLR4	The highest	Bacteria-induced myocardial injury; virus-induced myocardial injury; contribute to myocardial inflammation; doxorubicin-induced DCM; MI; myocardial ischemia-reperfusion injury; higher expression in patients with chronic HF	Inhibition of TLR4 is beneficial for the progression of HF; blocking molecular such as the statin family, eritoran, RP105, and ghrelin

TLR9	The lowest	Bacteria-induced myocardial injury; myocarditis; DCM; contribute to myocardial inflammation; diastolic HF; elevated mtDNA in HF patients	Inhibition of TLR9 is beneficial for the progression of HF; pretreatment with synthetic TLR9 ligand
